# Lifetime Abuse and the Barriers to Women Aged 50 and Over Using Domestic Violence Services in Germany

**DOI:** 10.1177/10778012241313481

**Published:** 2025-01-28

**Authors:** Grit Höppner

**Affiliations:** 1 Department of Social Work, Catholic University of Applied Sciences North Rhine-Westphalia, Münster, Germany

**Keywords:** domestic violence, age-sensitive support, intersectionality, age/aging, gender

## Abstract

Women often suffer abuse for many years before they turn to social services for victims and survivors of domestic violence (DV). This article examines the barriers that prevent women aged 50 and over from using these services. Adopting an intersectional approach, the article presents findings from a qualitative research project conducted in Germany. The findings suggest that services designed for DV victims and survivors in mind have not succeeded in eliminating these barriers to older women accessing the services. This calls for age sensitivity so that DV services can actually provide support to DV victims and survivors of all ages that they claim to offer.

Lifetime abuse is a multifaceted phenomenon that is defined in different ways according to discipline and theoretical approach. The article defines lifetime abuse in line with Hamby et al. (2016), who introduce a life course and poly-victimization dimension to the definition of elder abuse in order to draw attention to both the cumulative burden of victimization and the need to overcome the specialization that is done in victimization research according to life stages and forms of abuse. Because the study presented in this article is conducted within a feminist framework, this definition is combined with an understanding of abuse and violence against women that is informed by the domestic violence (DV) movement (Brandl, 2000; Saltzman et al., 2002; UN Department of Economic and Social Affairs, Division for Social Policy and Development, 2013). Lifetime abuse thus covers sexual violence, physical violence, and psychological abuse over the life course by a woman's current or former spouse or intimate partner. The study findings of this article demonstrate that an intersectional perspective that highlights the power relations of age and gender adds two further perspectives to this definition of lifetime abuse that are relevant to an analysis of barriers to DV services in Germany (Hagemann-White, 2016). First, focusing on the power relations of age helps us to better understand why older women use DV services less often than younger women (Federal Ministry for Family Affairs, Senior Citizens, Women and Youth, 2012b). Second, focusing on the power relations of gender helps to identify care relationships and ideas of good motherhood that may not only prevent women from using DV services but also suggest that the definition of lifetime abuse should be extended to include adult children as potential perpetrators. This understanding of lifetime abuse goes hand in hand with empirical findings that gender-based abuse and violence affect women more frequently in their social relationships than they do men (Federal Criminal Police Office, 2021; Federal Ministry for Family Affairs, Senior Citizens, Women and Youth, 2012a, 2014).

The second women's movement in Germany in the 1970s already made it clear that abuse can have serious consequences for DV victims and survivors (Brückner, 2018; see also Stöckl & Penhale, 2015). DV services in Germany have therefore developed diverse structures to support women with different needs. These services provide short-term support in emergencies and long-term support in coping with one-time, repeated, or lifetime abuse. In addition, these services provide preventive support for girls and younger women, including educational trainings in schools about self-defense. Services may be residential (e.g., short- and long-term shelters) or non-residential (e.g., counseling, legal and other services provided by agencies and community-based services). Nevertheless, women's shelters and specialized counseling centers are used by only about one third of women experiencing violence and abuse (European Union Agency for Fundamental Rights, 2014). The composition of the users of DV services in Germany shows that these services do not reach all women equally: Women aged 50 and over, women of all ages in the middle and higher education and income classes, and women of all ages with disabilities are significantly less likely to use these services. Statistics on the number of women living in shelters (Frauenhauskoordinierung e.V., 2022) show that in 2021 almost three quarters of DV shelter residents were aged between 20 and 40 years (71%) and only a small proportion were 50 years or over (7%). This finding was confirmed by the Federal Ministry for Family Affairs, Senior Citizens, Women and Youth (2012b, 2014) in relation to specialized counseling centers. I conclude that the reason why fewer women aged 50 and over use DV services is not that they need these services less than other DV victims and survivors, but that barriers make it difficult for these women to use these services. This is also the conclusion of Helfferich and colleagues, who point out that access thresholds can inhibit the use of women's shelters, specialized counseling centers, and other DV services provided by professionals in Germany (Federal Ministry for Family Affairs, Senior Citizens, Women and Youth, 2012a). This discrepancy is addressed in the study presented here, which focuses on older women victims and survivors of DV. The central question of the study is: What barriers prevent women aged 50 and over from using DV services?

An intersectional analytical framework is particularly well suited to answer the research question and to examine the importance of age/aging and gender in preventing women aged 50 and over from using DV services. Age/aging is both a *state* (i.e., a person is assigned a certain age) and a *process* (i.e., aging progresses continuously over the life course) (Höppner & Wanka, 2021; van Dyk, 2015). Gender is defined as a socio-cultural phenomenon and not as a given, natural fact or an individual characteristic of a person (Ehlert, 2020). An intersectional approach allows for an analysis of how the intersection of age/aging and gender contributes to the emergence of barriers. It thus allows for a multilevel analysis (Winker & Degele, 2009) that includes several levels that are particularly relevant to the emergence of barriers, namely those of structure, representation, and identity. While intersectional analyses have been applied to an understanding of older women victims and survivors of DV and barriers to obtaining the services they need in Latin America (Bonilla, 2021), the UK (Finnegan, 2022), and Canada (Etherington & Baker, 2016), this is the first intersectional analysis of barriers in Germany. It also incorporates a process-oriented perspective into the intersectional framework by providing additional information on whether barriers remain constant or change.

The following section introduces the intersectional framework in more detail. The article then presents the qualitative research design used to analyze interviews with DV victims and survivors and social workers, the websites of DV services, and empirical findings on barriers to accessing these services. Practical implications for reducing these barriers and suggested implications for providing age-sensitive support to women aged 50 and over will follow. Recommendations for further research in this area are provided. The conclusion summarizes the key findings of the intersectional analysis of barriers that prevent women aged 50 and over from using DV services.

## Intersectional Framework

Relations of social inequality have long been addressed and studied in the social sciences (Dederich, 2022). However, related research questions and established research contexts, such as gender, postcolonial, queer, or disability studies, have focused on one main difference (e.g., gender, skin color, sexual preference, disability) and have tended to analyze and conceptualize it independently of other categories of difference. Although it is well known that relations of inequality are interrelated, this knowledge had not been developed into a systematic research framework until the term “intersectionality” was coined by Kimberlé Crenshaw in 1989 (Crenshaw, 1991; for an overview, see Dederich, 2022).

Intersectional approaches assume that relations of inequality cannot be analyzed through the use of one category or via the addition of several categories (Crenshaw, 1991; Knapp & Wetterer, 2003; Lutz et al., 2010). Instead, the intersections of multiple categories can help us better understand relations of inequality and the consequences of these categorical entanglements in terms of the distribution of life chances and people's social status (Biele Mefebue et al., 2022). This means that intersecting differences either open or close educational and socioeconomic processes as well as opportunities for participation (e.g., access to DV services), the allocation or denial of resources (e.g., professional help), and the attribution of identities (e.g., DV victims and survivors as recipients of professional help). The intersection of categories of difference can lead to multiple forms of discrimination and create multi-dimensional inequality (Dederich, 2022).

Winker and Degele (2009, p. 25) developed the analytical framework of “multi-level analysis” based on a capitalistically structured society with the dynamics of economic profit maximization to systematically consider categories of difference that are interdependent and represent levels of structure, representation, and identity. This intersectional framework allows for both an analysis of intersecting categories from a horizontal perspective (such as the intersection of gender and age) and an analysis, from a vertical perspective, of the different levels of social inequality that derive from this intersection (such as the macro, mezzo, and micro level).

The macro level of social structures may include the intersection of heteronormativity and ageism, among others, as manifested through institutions and laws. At the mezzo level of cultural symbols, the intersectional framework helps to explore how social norms and values are discursively linked to the processes under analysis and to elaborate how “security frictions and justifications for inequalities” are constituted (Degele & Winker, 2007, p. 12). At the micro level, the focus is on interactively produced processes of identity formation and subjects’ experiences, leading to an analysis of “identity constructions as strategies for coping with uncertainties” (Degele & Winker, 2007, p. 12).

These three levels of analysis are further elaborated by Ehlert (2020). Ideas about different dimensions of gender can be used to analyze the ways in which society is structured so that people of a certain age and gender experience barriers to the use of DV services. Like Winker and Degele (2009), Ehlert refers to the structural level, interpreting society as a complex context in which the structure is shaped by gender. This structure is materialized in the living conditions of women and men (e.g., family care duties are often associated with femininity), in the help provided by social services in Germany (e.g., DV services are primarily directed at women and hardly ever at men), and in other ways. To answer the research question of the present study, the interviews with DV victims and survivors and social workers were analyzed to understand whether *power relations of gender and age/aging* create barriers to the use of DV services.

At the representational level, Ehlert refers to the concept of “doing gender” (West & Zimmerman, 1987, p. 125). She refers to gender as a social construct that is reproduced in interactions, requires gender knowledge, and serves as a feature of gender difference. Gender as a social construct is illustrated by the concept of the “power to violate” (*Verletzungsmächtigkeit*) and the “openness to violation” (*Verletzungsoffenheit*). Here, the construct of masculinity portrays the male body as having the power to harm and that of femininity portrays the female body as being open to suffer harm (Wobbe, 1993, p. 113—116). The interviews with DV victims and survivors and social workers were analyzed to determine whether *gender and age/aging as social constructs* might contribute to barriers to DV services. At the representational level, Ehlert also refers to gender as an effect of language and social debates that are condensed in gender discourses. They can, for example, contribute to the creation of a link between femininity and fear in the context of violence (Mehta, 2010). To answer the research question, the websites of DV agencies and interviews with DV victims and survivors and social workers were analyzed to determine if and how *gender and the age/aging effects of discourses* might contribute to the creation of barriers.

At the identity level, Ehlert defines gender as a dimension of conflict arising from normative expectations and ideas of femininity and masculinity on the one hand, and gendered identity formations and life-history developments on the other. The conflicts that DV victims and survivors are dealing with can be seen in how they deal with and end intimate partner violence and thus develop their own gender identity (see, e.g., Baly, 2010), for example, from the idea of femininity as being open to suffering harm to the idea of femininity as being safe from harm. The interviews with DV victims and survivors and social workers were analyzed to learn more about the *conflicts* that women aged 50 and over perceive when experiencing abuse, coping with insecurities, and thinking about using DV services.

Although barriers to services have been analyzed before, it is only in recent years that the number of analyses about age has increased (e.g., Beaulaurier et al., 2007; Crockett et al., 2018; Gill, 2022). This article contributes to the analysis of barriers by using an intersectional framework of age/aging and gender. The intersection of age and gender allows us to analyze whether women aged 50 and over experience barriers to using DV services because of their age and gender. The intersection of aging and gender allows for an analysis of both barriers from a life course perspective of older women victims and survivors of DV and access thresholds for older women victims and survivors of DV compared to younger DV victims and survivors. In doing so, this study responds to Hamby et al.'s (2016) call to overcome the specialization of victimization research based on life stages and instead to use a life course perspective (e.g., Band-Winterstein & Eisikovits, 2014; Herrenkohl et al., 2022).

## Methods

In 2022, three research assistants and I conducted a qualitative study in the western part of Germany. The aim was to learn more about the barriers that prevent women aged 50 and over, women in the middle and higher education and income classes, and women with disabilities from using DV services. Three types of qualitative data allowed for a multi-perspective analysis of the barriers to using of DV services.

### Participants and Data

As a type of qualitative data, expert interviews (Meuser & Nagel, 2009) with social workers were conducted and analyzed to answer the research question and to take into account the perspectives and perceptions of barriers that women aged 50 and over may experience from professionals working in different types of DV service facilities (i.e., general women's counseling center, specialized sexual violence center, women's shelter), with different funding structures (i.e., autonomous, confessional, state), and with specific tasks (counseling and support of women at risk of violence or other DV victims and survivors, public relations). Ten experts were selected based on these specific characteristics. They had between six and thirty years of professional experience in these nonresidential and residential DV services.

As a second type of data, the websites of the 10 facilities in which these social workers are employed in were analyzed. The statements made by the social workers and the findings of the website analysis are considered in the result section if they refer to the intersections of age/aging and gender and to power relations, constructs, effects of discourses, or categories of conflict.

To ensure a balanced number of interviewees and a heterogeneous sample, 10 expert interviews (Meuser & Nagel, 2009) with women who had experienced violence and abuse in the past were conducted and analyzed as a third type of qualitative data. These women are defined as experts on their own life worlds. This idea follows an important principle of understanding addressed in social work (Thiersch, 1992). The women interviewed met the following criteria: They were aged 50 or over, and/or they were from the middle or higher education and income classes, and/or they self-identified as having a disability. See Table 1 for the criteria met by the interviewees:

**Table 1.** Sample of Interviewed Women Who Have Experienced Abuse and Violence (Author's Illustration).

	Form of abuse and violence experienced, duration	Age	Education and income class	Disability
1	Psychological abuse by former partner (several years), sexual abuse in childhood	53	Degree, unemployed	Chronic pain
2	Psychological abuse (4–5 years), physical violence (1 year) by former partner	50	High school diploma, employed	—
3	Psychological and physical abuse and violence (22 years) by former partner	60	High school diploma, employed	Disability due to breast cancer in the past
4	Psychological and physical abuse and violence (6 years) by former partner	56	High school diploma, employed	—
5	Psychological abuse by former partner (2.5 years), physical and psychological abuse in childhood	37	High school diploma, employed	—
6	Psychological and physical abuse and violence (8 years) by former partner	35	High school diploma, employed	—
7	Psychological, physical, and sexual abuse and violence (2 years) by former partner	52	High school diploma, employed	—
8	Psychological and physical abuse and violence (2.5 years) by former partner	30	High school diploma, unemployed	Physical disability since childhood
9	Psychological and physical abuse and violence (2.5 years) by former partner	60	Degree, employed	—
10	Psychological and physical abuse and violence (2 years) by former partner	30	Degree, employed	—

In order to be able to analyze the barriers that prevent women aged 50 and over from using DV services, the statements of the six women aged 50 and over are considered in the result section, as well as the statements of the four younger women, if they relate to these barriers and the intersection of age/aging and gender. A mixed age sample helps to understand the intersectional idea of age/aging and gender as being central categories of difference across the life course. Hamby et al.'s (2016) call to move beyond life-stage specialization in victimization research by adopting a life course perspective is considered here to offer explanations as to why older women are less likely to use DV services than younger women do. In line with the research question, the analysis focuses on barriers that prevent women aged 50 and over from using DV services.

### Sampling

Existing network structures in the western part of Germany were used to select the sample of professionals using the self-activation approach (Reinders, 2012, pp. 119–120) and according to the criteria mentioned above. The social workers were provided with information about the study via email and asked to participate; all but one responded positively. The 10 interviews were conducted via telephone or video call. The interview guide consisted of three parts: (1) the facility's target group, reasons for women using the DV social service, women's journey to the social service; (2) barriers to the social service system that women aged 50 and over may experience from the perspective and perception of professionals; (3) developing new DV social services for at-risk or battered women aged 50 and over, women in the middle and higher education and income classes, and women with disabilities.

The websites of the facilities in which the 10 social workers are employed were analyzed, with the focus on how the facility, professionals, services offered, those who use the facility, as well as their life worlds and social problems, steps in the process of providing assistance and support, and barriers to the use of this social service that are mentioned (e.g., lack of accessibility) are presented.

Contact with DV victims and survivors was made through social workers who acted as gatekeepers (Reinders, 2012, pp. 118–119); one woman contacted me directly. Due to the sensitive interview situation and the risk of re-traumatization, it was particularly important to adhere to certain principles of research ethics. These include voluntary participation, the possibility to break off the interview at any time, interviews being conducted in a facility in which a professional can be called in at any time to provide support, and the protection of the participants by means of the anonymization and secure storage of the data (Helfferich et al., 2015). The study is part of the WIN_innovation program of the Catholic University of Applied Sciences North Rhine-Westphalia, Germany, which in turn is part of the German federal-state program “FH Personal” (funded by the German Federal Ministry of Education and Research). The study was reviewed as part of an institutional review board process at the Catholic University of Applied Sciences North Rhine-Westphalia, Germany, and in the context of the ethical standards of the WIN_innovation program.

The interview guide for the 10 women consisted of four parts: (1) knowledge about Germany's social service system, reasons for using specialized DV services, how to access these social services; (2) experience of, evaluation of, and barriers to the social services; (3) impressions of how the social services are presented to the public; (4) reflections on their own decision to use DV services.

### Data Analysis

The transcribed and anonymized interviews and the websites were analyzed using qualitative content analysis according to Mayring (2000, 2015). This method provides a systematic, rule-based qualitative text analysis and uses principles such as categories and step models. The following two main procedures of qualitative content analysis are applied in this research:

1. The deductive application of categories means that the researcher works with previously formulated, theoretically derived aspects of analysis and uses a methodologically controlled assignment to a text passage (Mayring, 2000). In this study, deductive categories for analyzing the 20 interviews were formulated based on the interview guide (e.g., knowledge of the German social services, reasons for using a DV service, access to the service) and the intersectional framework (e.g., structural level: age and gender relations; structural level: aging and gender relations). Deductive categories for the analysis of the ten websites were formulated based on the analysis grid presented above.
2. The inductive development of categories follows the idea of formulating a criterion that determines the aspects of the material to be analyzed. Following this criterion, the researcher has to work through the entire material and derive categories in a step-by-step process. Through the use of feedback loops, these categories are revised or reduced to main categories and tested for reliability (Mayring, 2000). This procedure was applied to the analysis of the interviews, after which the inductive categories of assessment processes, familiarization, and attitude were formulated, among others.

In order to achieve rigor and establish the credibility of the study and its findings, the research team reflected both on how to conduct the interviews (e.g., openness to interviewees) and on how to formulate the deductive and inductive categories. I presented and discussed the findings at various national and international conferences, and feedback was incorporated into updates of the study.

## Results

### Barriers to the Use of DV Services

The analysis revealed several barriers that prevent women from using DV services. Some of these barriers affect women regardless of their age, such as ambivalence about leaving an abusive partner and not knowing exactly what support professional help can provide. Some barriers are related to categories other than age/aging and were therefore not included in this analysis.^1^

In order to identify the significance of age/aging and gender as power relations, constructs, effects of discourses, and categories of conflict that can prevent women aged 50 and over from using DV services, the findings are organized according to the following four deductive categories, formulated in accordance with Winker and Degele's (2009) intersectional framework and Ehlert's (2020) definition of gender dimensions:

- Structural Level: age/aging and gender power relations,
- Representational Level: age/aging and gender constructs,
- Representational Level: age/aging and gender effects of discourses,
- Identity Level: age/aging and gender as categories of conflict.

The inductive method of developing categories that emerged from the qualitative analysis of the data revealed themes for both the intersection of aging and gender (familiarization, awareness, attitude) and the intersection of age and gender (structural dependencies, classifying one's own experience as abuse, how DV services address DV victims and survivors, coping, beliefs). These findings are presented as subcategories of the four deductive categories. In addition, the inductive method of category development revealed the assessment processes of evaluation, classification, consideration, and decision as categories relevant to both intersections. These are presented throughout the analysis.

### Structural Level: Age/Aging and Gender Power Relations

In terms of the process of aging, it is relevant that women acquire standards during their lives that help them evaluate behavior in social relationships and distinguish abuse from behavior that is considered normal. For example, a 60-year-old woman stated that her parents belonged to the generation “that grew up during the war” and “abuse within the family was considered normal … my father slapped my brother when he was naughty”; for this woman, violent behavior was part of parenting and affected her understanding of partnership behavior in general and the behavior of her abusive partner in particular. One social worker reported that, in her professional experience, older women often experienced traditional practices in partnerships in which violent behavior was an element which these women became *familiar* with and which they found normal. This is reflected in the applicable legal framework, given that marital rape only became a criminal offense in Germany in 1997. One social worker worked with older women who used DV services and who had experienced many years of abuse in their marriages:

I can remember a woman who actually came to us with two broken arms, so it was over for her and the husband had the onset of senile dementia with increasing aggression. … That was because she feared for her life. She spoke of decades of abuse by her husband … but she could control that, that he had hurt her, but that it had never been that bad. … In that situation he came at her with a broken broomstick and wanted to hit her over the head and she really had the impression that he wanted to kill her. … [S]he hadn’t experienced that before. She said ‘It was always terrible, but I learned to live with it,’ and at that moment it was over and she had no control anymore and then she left.

The decision to leave the abusive partner was based on the increased intensity of abuse and the woman's loss of control in this situation. Learned behavior and intimate partner and relationship norms influenced her evaluation of an act as either non-abusive or abusive. The evaluation could have become a barrier if the husband's abusive behavior had been within the usual range of actions. Once a partner's actions exceed the previously experienced intensity and the woman's subjective coping behavior no longer suffices, this can lead to a reevaluation of the husband's behavior.

In terms of age, both the abused women and social workers highlighted the women's *structural dependency* on their abusive partner or other family members. One barrier is financial dependency on one's partner, which can prevent older women from ending a violent partnership due to the “fear of falling into poverty in later life.” The prospect of a financially secure later life may then be considered more important than that of an abuse-free later life.

Another barrier resulting from structural dependency is when women are dependent on their children. These conditions can be further subdivided into two categories. In one category the children are not yet living independently and they experience domestic violence. Only when the children have left home do the mothers separate from the abusive spouse:

We often had women who came once the children had left home—women who really still had the attitude that “[a]s long as the children are there, I have to put up with it.” And when the children left home, because they were now standing on their own two feet, they said, “That's it, I’m leaving the next time he attacks me.”

The idea of a good caring mother who accepts her partner's abuse to enable the family live together is a gender-specific construct and a common idea of femininity. One social worker noted that younger women often leave their partners quickly if their children witness abuse or if their partner is abusive to the children. Thus, “staying for the sake of the children” is more likely to be an attitude held by older than by younger women. One younger woman justified this attitude by saying that she was afraid that her child would witness the father's abusive behavior and that social services might take the child into care.

In a different category, the abusive person in a DV situation is not the spouse but an adult child. One social worker spoke of a 70-year-old woman who was living with her grown-up daughter and her family after the older woman's spouse had died:

She lived with her daughter and her daughter got remarried and, in this house, there were separate rooms where she could live by herself. And the daughter's husband was constantly in conflict with his mother-in-law, with this older lady. And this increasingly escalated, there were physical altercations, arguments and in the winter months, for example, the electricity or heating was switched off so that she could not heat her rooms. And then the daughter gained access to her bank account and ultimately took away her income.

When the woman contacted a DV service, she was “completely in despair” and was “very emotional about it, completely shaken by her daughter's behavior—that she should have stood by her husband like that.” The older woman's idea of a good child was not compatible with her daughter's behavior, which she found deeply unsettling.

Another example of this category is an informal care setting at home. Here, too, the difference between non-abuse and abuse plays a role, especially when women have not yet developed a standard of evaluation because they have nothing to compare their current situation to. For example, one social worker spoke of an “older lady” and her son, who was her caregiver:

[The son] simply came into [his mother's] apartment and stole things or even decided things against her will. … And she was also afraid that her son would have her declared incapacitated. … They often doubt their own judgment: “Is this normal or is it abuse?” “Is he allowed to do that?”

This woman's daughter, who lived in a different city, contacted a DV counseling center so that social workers would make it clear to her mother that her brother's behavior constituted abuse. In this family, care and abuse are distributed according to widespread gender-specific patterns. However, because of the informal care setting, it is often difficult to reduce such dependencies because not all women who are living in informal care settings have access to information about support services or they might not have barrier-free access to websites.^2^ That is, even if these women have chosen to find out about DV services, they might face barriers. Furthermore, even if women then decide to use these services, they might face barriers due to facility-related features, for instance, the lack of an elevator.

The study confirmed Helfferich et al.'s findings (Federal Ministry for Family Affairs, Senior Citizens, Women and Youth, 2012a) that while DV services can be used by women of all ages, older women use these services less often. While the social workers recalled many younger women who used their DV service, most of them hardly recalled any older women who did so. One social worker said that she and her colleagues had not yet considered providing services for older women because of limited personnel capacities:

We have wisely ignored this because we were unable to estimate what kinds of new problems we’d be facing, not least because of the personnel resources available. Nevertheless, I assume that there is a great need for this.

DV services in Germany were established as one of the results of the second women's movement in the 1970s. Back then younger women fought for DV services and shaped them by focusing on women of reproductive age. Although the women of the second women's movement have aged, the DV services they fought for and shaped still seem to be directed at younger women. The intersection of aging and gender helps us to understand how *age-specifically* the system of DV services is structured in Germany.

To conclude, age/aging and gender power relations can influence all four assessment processes that DV victims and survivors go through: during the process in which women evaluate their partner's or children's actions to decide whether they are non-abusive or abusive; during to process of judging whether or not to classify their own experience as abuse; during the process in which they consider whether they would like to get professional help; and finally, during the process of using DV services.

### Representational Level: Age/Aging and Gender Constructs

Male constructs were highlighted that finally led two women to DV services. The 70-year-old woman mentioned in the above experienced a long-term violent partnership that enabled the couple to reproduce *gender differences* through the husband's “power to violate” (*Verletzungsmächtigkeit*) and the woman's “openness to violation” (*Verletzungsoffenheit*). Not only was the husband able to determine the construct of masculinity by using his male body as a powerful medium to inflict injury, but the woman reproduced ideas of femininity by presenting her body as open to injury (Wobbe, 1993). However, the husband developed dementia in later life and engaged in increasingly abusive behavior, which the woman was no longer able to control. She thus separated from her husband and used a DV service.

In another case, a son had been violent toward his mother from an early age. As an adult, the son did bodybuilding and took steroids. As an 80-year-old woman, the mother could no longer stand the son's abusive behavior and broke off the violent mother–son relationship. The son had been taught from an early age by his father “that women were just not worth much and that you are allowed to push them around,” and the social worker who talked about the case concluded, “that's what her son did at some point.” Thus, constructs of masculinity and femininity can prevent older women from using DV services if they are maintained throughout a partnership or a mother–son relationship. It was only when the previously known intensity of the husband's or son's behavior increased that these women were able to end the relationship and use DV services, the effect of which was that they no longer reproduced these gendered constructs.

Age is a relevant factor in constructs in which specific forms of abuse are linked to a woman's age. For example, some of the social workers talked about age-specific forms of abuse, stating that physical and sexual abuse was linked more to younger than to older women. As one social worker puts is:

And especially when they are older, we know that physical and sexual abuse decreases, and psychological abuse increases in abusive relationships, so that an abused women might think, “Well, that's not as bad as it used to be.”

One social worker problematized the link between sexual abuse and *age images*: “[T]he image is actually always that it is young women who are raped. But older women are just as frequently affected.” The image of the young, desirable woman in contrast to the older, “asexual” woman (Crockett et al., 2018) seems to still be powerful in myths about sexual abuse. This image influences older women's decision to seek the help of DV services: “This image, it concerns only young women and now that I’m 65 and I allow myself to talk about it” can prevent older women from using a DV service. We are faced here with a double life course perspective: Younger women, the main target group of DV services, are helped to cope with abuse and violence and are offered preventive services to help them avoid abuse and violence in later life. By contrast, women aged 50 and over use DV services less due to the prevalent age images and based on their own assessment that the abuse and violence they experienced at a younger age was more intense than the abuse and violence they are experiencing now. Thus, they might often see no reason to use a DV service in their current situation.

To conclude, the intersections of gender and age/aging can influence the ways in which women evaluate and perceive abuse. This, in turn, can influence whether women classify their partner's or child's actions as abuse and, further, whether they consider seeking professional help.

### Representational Level: Age/Aging and Gender Effects of Discourses

Aging is relevant in one interview with a woman aged 60 who experienced abuse and violence in her intimate partner relationship for 22 years. In retrospect, she stated that it would have helped her in her deliberations as to whether to use DV social services if the abuse of women in intimate partner relationships had been *discussed in public* as much then as it is today. Then she may have been aware of the problem as a young woman and it may have been easier for her to consider using DV social services in the course of her abusive partnership.

Classifying one's own experience in an intimate partner relationship, family, or informal caregiving relationship as *abuse* can still be challenging for women aged 50 and over. The websites that were analyzed apply the terms “abuse” and “violence” to their physical, psychological, sexual, and sometimes social and economic forms. Some of the women pointed to a discrepancy between how their individual experience fit with the schematic concepts of abuse and violence. First, several women problematized the fact that before using DV services they had understood abuse and violence to mean physical actions, and they only expanded this understanding during conversations with social workers. They problematized the fact that the concepts of abuse and violence suggest which physical actions must have been experienced in order to be able to describe a certain behavior as abusive and violent. If women have experienced hurtful actions that are not defined as abuse or violence as they understand the terms (e.g., psychological abuse), these women may not be able to perceive themselves as the victims of abuse. Some women expressed uncertainty as to whether their experience was the “right one” to be addressed by DV services. According to one social worker, older women often wonder “Am I in the right place here? Have I experienced abuse at all?” and “[Is] my problem even big enough” to be eligible for DV social services? To avoid the feeling of uncertainty, one woman suggested defining abuse and violence in terms of the consequences of the behavior they had experienced. According to one social worker, a broader understanding of abuse and violence could help to incorporate forms that have not yet been covered by the concept, such as neglect and psychological abuse by an adult child who is providing informal care in the home. Up to now, these forms of abuse have not been addressed by most agencies providing DV services in Germany. In addition, one social worker highlighted the problem that rape is associated with age images. Thus, older women might feel unsure about whether they have in fact experienced sexual abuse.

Another age effect of discourses becomes apparent through the analysis of DV services websites. The analysis of 10 websites showed that DV services do not appear to reach out to all women equally, because women are more likely to be *addressed* as younger women and middle-aged women. By naming “discos,” “pubs,” “the internet,” “school,” or “the workplace” as potential places where abuse and violence occur, and by referring to “parents,” “partners,” “friends,” and “teachers” as people who can provide support, women are addressed as pupils, students, and employees. They are also addressed as mothers of young children by referring to childcare options during counseling and service relating to switching kindergarten and school. While the photos on the websites of organizations providing DV services show women of different cultural backgrounds and appearance, the various ages of those using a service are not usually in evidence. Only one website shows a woman using a walker and another one using a wheelchair. Women in informal care contexts are not portrayed even though they, too, can be DV victims and survivors. If they want to find out information about DV services online, the websites may lead to them feeling they are not being addressed.

To conclude, when it comes to the effects of discourses, the fact that discourses are limited to the lack of publicity about DV when those women who are older today were young and the fact that DV services today focus on younger women as a target group can make it difficult for women aged 50 and over to classify their own experience as abuse and to consider whether they want to use DV social services.

### Identity Level: Age/Aging and Gender as Categories of Conflict

The interviewees mentioned several conflicts arising in women aged 50 and over on account of normative expectations and subjective experiences. The process of aging is relevant in relation to women's *attitude* to the use of DV social services. One 60-year-old woman spoke about her mother's Christian influence. Her mother belongs to the generation, which encompasses the idea that women should sacrifice themselves out of love for their children and put up with abuse in a partnership. This interviewee had internalized this as an ideal of femininity while she was growing up. On account of holding this attitude she only left her abusive partner once her children had left home. She problematized the idea of keeping private matters private, which is closely related:

Because I’m of an age that parents raised you accordingly, you don’t tell the outside world. It's all peace and joy. In this form, if you always have that in mind, you first think you’re to blame, and I think you can only hope that this generational problem will be solved at some point.

One social worker observed that it is particularly difficult for these women to contact DV services because their attitudes pose a barrier: “Often when women arrive here, there are also many older women [who say] ‘I would never have thought that I would ever need such help’.” The feeling of shame is particularly strong in these women because they think they have failed at being a good wife or a good mother, and it is a kind of disgrace to accept external help. The social worker pointed out that these women understand abuse and violence as an individual failure, not as a societal problem.

Age is also relevant when it comes to strategies for *coping* with abuse and violence:

That is more of a mental barrier; younger women are much more likely to say ‘Yes, I need help or support.’ Or they’re just much more open to it, I think that certainly has something to do with age and with dealing with social or psychosocial problems.

At a time when there is war in Europe, memories of wartime rape can resurface more strongly in older women. According to one social worker, the internal conflicts older women experience often result from their *belief* that the sexual abuse happened too long ago and there is no point in getting help. In conclusion: Both age and aging can be barriers when women consider whether or not to seek professional help.

## Discussion

**Figure 1.**
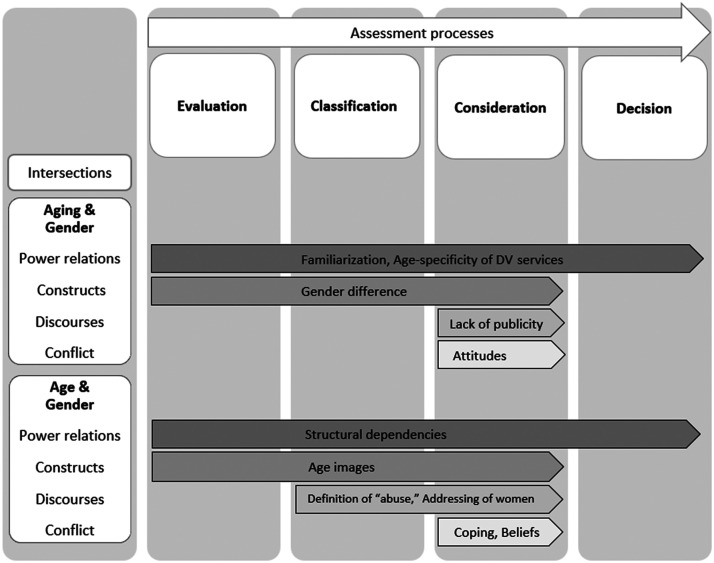
Assessment processes of women aged 50 and over and barriers to the use of DV services (author's illustration).

The aim of the present intersectional analysis is to examine the barriers that prevent women aged 50 and over from using DV services. The significance of the intersections of age/aging and gender at the levels of structure, representation, and identity in relation to power relations, social constructs, effects of discourses, and categories of conflict were examined in terms of how they contribute to creating barriers to using DV social services. The analysis showed that not all identified barriers are constant and that some vary. A process-oriented perspective led to the identification of four assessment processes when women experience abuse and violence, processes in which women aged 50 and over evaluate whether their partner's or child's actions are abusive or not, reflect on whether to classify their own experience as abuse, consider whether to seek professional help by weighing the advantages and disadvantages, and, finally, decide to use DV social services. In the empirical analysis, these four assessment processes were linked to the intersections of aging (as a process) and gender, and age (as a state) and gender, and barriers were identified. The main findings are summarized in Figure 1 (assessment processes and intersections [white background] and barriers [grey background]).

An important finding is that barriers vary depending on the assessment phase a woman is in. To make recommendations on how to reduce barriers, it is important to consider each barrier in the context of the specific assessment phase.

### Recommendations for Reducing Barriers According to Assessment Phase

In the evaluation, classification, and consideration phases, the familiarization with abuse, gender differences, structural dependencies, and age images can be particularly important in preventing women aged 50 and over from using DV services. Therefore, information about age, gender, education, and intimate partner relationship norms, information about structural dependencies and norms for classifying behavior as abuse and one's own experience as abuse, and information about available DV services is needed (Brandl, 2016). These findings confirm that there is a lack of knowledge and awareness about abuse in social relationships in later life. For example, abuse may occur for the first time as a result of changes in the health of a woman's spouse (Band-Winterstein & Avieli, 2019; Görgen, 2010), and an adult child or grandchild may commit abuse against the mother or grandmother (Smith, 2022). Therefore, it is important to provide information and raise awareness about the risk of abuse for older women. In informal care settings, this can be done through training of caregivers and through the medical services provided by health insurance companies.

Other barriers, such as attitudes, coping strategies, and beliefs held by women aged 50 and over, are particularly strong during the consideration phase. This phase is particularly sensitive because most barriers arise as these women weigh the advantages and disadvantages of using DV services. The findings suggest that, when services address DV victims and survivors, they should consider age sensitivity to reach women of all ages and to help them reflect on their attitudes, coping strategies, and beliefs, and raise awareness about violence. To address facility-based barriers that may prevent women from using DV services at the decision-making phase, DV services could be expanded to include proactive counseling.

In this study, age/aging and gender power relations were found to be particularly relevant in each assessment process. This finding underscores the importance emphasized in gender studies of not individualizing violence, but rather considering its embeddedness in the structure of power relations (Hagemann-White, 2016). Thus, a woman's decision to use DV services is not a private one. Rather, it depends on many factors, such as her parents’ parenting style and the attitudes it fosters about how to deal with abuse in the family, ideas about femininity, age images, and facility-based barriers. This finding enriches concepts that illustrate the barriers to seeking help faced by women aged 50 and over who have experienced intimate partner violence. For example, Beaulaurier et al. (2007) developed such a concept of barriers to help-seeking, focusing on internal factors (e.g., self-blame, family protecting), perpetrator behavior (e.g., isolation, jealousy), and external factors (e.g., family, clergy, justice system, community). The findings of this study expand on this concept by invoking the barriers reproduced by DV social services themselves, as well as age/aging and gender power relations.

All these recommendations might help women aged 50 and over to use DV services and they may support DV victims and survivors to report abuse and violence to the police. This is important to minimize the presumed high number of unreported cases of violence against women, which could be multiplied by the number of unreported cases of abuse in later life (Gröning & Yardley, 2020). It is important to include women of all ages in the samples of representative studies to minimize the number of unreported cases.

### Recommendations for Reducing Barriers in the DV Service System

In the interviews that were conducted, social workers stated that they were aware that women aged 50 and over were less likely to use DV services than younger women. To reduce barriers, they considered new ways to reach these women in a timelier and more responsive way in order to minimize barriers for women who have not yet used DV services. Some of the social workers have developed creative approaches, such as renting an accessible room to counsel mobility-impaired women. However, the burden of providing comprehensive and age-sensitive support should not be left to social workers alone. Rather, it requires structural changes in the DV service system, such as an increase in financial and human resources. According to the principle of solidarity, these resources must be distributed equally among DV victims and survivors of all ages. An intersectional analysis, which has the capacity to expose discrimination, shows that some DV services have not yet been able to address the unequal position of women aged 50 and over by providing support when they experience abuse, but have in fact reproduced these barriers in their own practice. The effect is a two-age class system in relation to DV services, as it seems to be easier for young and middle-aged women to access the system and more difficult for older women to do so. Social workers in the shelters included in this study in particular argued that the difference between younger and older women is caused by a lack of resources that would prevent shelters from creating a lower threshold for older women. However, these social workers help to maintain an age-coded system that might exclude them if they wanted to seek professional support. The high proportion of female social workers in DV services statistically increases the likelihood that they themselves will experience abuse.

The relevance of power relations highlights the value of a definition of lifetime abuse that takes into account the structural conditions and dependencies that make abuse possible over the course of a lifetime. Expanding the definition of lifetime abuse as poly-victimization embedded in age/aging and gender power relations helps us to better understand the barriers that prevent women aged 50 and over from using DV services. Not only the nature of DV services in Germany and their focus on younger DV victims and survivors, but also the limitation of perpetrators to the current or former spouse or intimate partner means that older women experiencing abuse or violence by an adult child are often not considered users of DV services and are thus marginalized in terms of access to these services. In contrast, the service system in Germany that provides support for people aged 60 and over usually does not have the expertise to work with older women who are DV victims and survivors who are not otherwise impaired. The central aim of these professionals should be to bridge the gap between these two systems of social support and to address the needs of women aged 50 and over in coping with their experiences of abuse and violence. Age-sensitive DV services that refuse to reinforce the vulnerable social position of women aged 50 and over can present a step forward in breaking down barriers.

### Recommendations for Further Intersectional Research in DV Services

A critique of intersectional approaches and analysis concentrates on the focus on class, race, and gender, and thus on a limited number of already well-researched categories. This selection is based on how intersectional approaches have developed over time, as they initially considered specific life situations and discrimination experiences of Black women. As a result, further categories that promote inequality are less frequently problematized, such as the categories of age and aging (Denninger & Schütze, 2017; Höppner & Wanka, 2021). In the intersectional analysis that is presented here, I counter this critique by considering age and aging and their intersections with gender. This theoretical framework allows for a richer analysis of barriers that arise as a result of age and during the aging process. The potential of considering such a process-oriented perspective in an intersectional framework is in its capacity to provide further information, for example, on how habits are formed over the course of a lifetime, the adoption of age and gender knowledge, and the appropriation of generational attitudes.

An analysis of barriers must include a critical reflection on life-stage–specific ideas and images associated with age, such as the association of rape with a younger age group and age-coded forms of abusive behavior (e.g., neglect) because otherwise these ideas and images are reproduced and reinforced (Crockett et al., 2018). This provides insights not only into the status of older women but also into that of younger women. A life course perspective that juxtaposes different ages challenges oversimplified attributions of behaviors to life stages and identifies processes of exclusion that begin when actors, actions, or instances of violence do not correspond to what is commonly associated with a particular life stage, such as rape in later life. As a result, the study demonstrates the value of the research field of violence against women incorporating a life course approach to violence and of gerontological research on elder abuse incorporating a gender analysis (Brownell, 2023; Crockett et al., 2015; UN Department of Economic and Social Affairs, Division for Social Policy and Development, 2013). The definition of lifetime abuse developed in this article is a step toward bridging these two strands of research, which have developed in parallel and, to all intents and purposes, in isolation.

In terms of methodical considerations, we can say that the sample of the study presented here is relatively small. However, Metcalf et al. (2018, p. 594) state that an intersectional approach can be used even with small samples: “[F]or too long in our quantitative work, researchers have prioritized statistical significance over meaning.” However, the findings and conclusions of this study should be tested for representativeness as part of a quantitative survey. As this study was conducted in the western part of Germany, the findings illustrate regional characteristics and the results cannot be generalized to the whole of Germany, although the structure of DV services in Germany is like that of the region examined in this study. A further study could use regional contrasting to show whether professionals and women aged 50 and over perceive similar barriers to those identified in this study. A further study could increase the number of older interviewees to include the perspectives of DV victims and survivors in their 60s, 70s, 80s, and older to learn more about barriers that prevent them from using DV services. A mixed-age sample allows for a life course perspective. However, researchers must avoid allowing one age cohort to speak for another. Finally, an approach other than the gatekeeper approach could be used in order to avoid pre-selection of interviewees by gatekeepers and reach women aged 50 and over who have had no previous contact with DV services to learn more about the assessment phase they are in.

## Conclusion

The intersectional analysis presented in this article promotes an intersectional multi-level analysis of the barriers identified through assessment processes that determine whether women aged 50 and over might use DV social services in Germany. The findings show that these women perceive age-specific barriers that have different relevance during the four assessment phases of evaluation, classification, consideration, and decision. Thus, an intersectional framework in social work research (Matsuzaka et al., 2021) helps to raise awareness of the need for analysis and DV services that are sensitive to differences and enables the development of recommendations that contribute to minimizing barriers to using DV services. Up to now, women aged 50 and over have been under-represented when it comes to domestic violence. The results of this study show that it is therefore important to bridge the gap between DV services and the service system that provides support for people aged 60 and over, as well as the gap between the research field of violence against women and gerontological research on elder abuse to support DV services in actually providing a support system for women of all ages.

## Notes

1. Class, for example, is relevant in the case of a woman whose spouse is a doctor or judge and her fear is that she will not be believed if she claims that she has been raped by that spouse. Disability is relevant when facilities do not provide physical assistance.

2. Three out of ten websites are readily accessible (e.g., possibility to enlarge the text and look up the meanings of words, read aloud function, sign language).
